# Cardiac manifestations in COVID‐19 patients—A systematic review

**DOI:** 10.1111/jocs.14808

**Published:** 2020-07-11

**Authors:** Ahmed M. A. Shafi, Safwan A. Shaikh, Manasi M. Shirke, Sashini Iddawela, Amer Harky

**Affiliations:** ^1^ Department of Cardiothoracic Surgery, Barts Heart Centre St Bartholomew's Hospital London UK; ^2^ Department of Medicine Queen's University Belfast Belfast UK; ^3^ Department of Respiratory Medicine University Hospitals Birmingham Birmingham UK; ^4^ Department of Cardiothoracic Surgery Liverpool Heart and Chest Hospital Liverpool UK; ^5^ Faculty of Health and Life Sciences University of Liverpool Liverpool UK

**Keywords:** cardiac biomarkers, cardiac manifestations, cardiovascular risk factors, clinical presentations, COVID‐19, SARS‐CoV‐2

## Abstract

**Objectives:**

The coronavirus disease‐2019 (COVID‐19) pandemic has resulted in the worst global pandemic of our generation, affecting 215 countries with nearly 5.5 million cases. The association between COVID‐19 and the cardiovascular system has been well described. We sought to systematically review the current published literature on the different cardiac manifestations and the use of cardiac‐specific biomarkers in terms of their prognostic value in determining clinical outcomes and correlation to disease severity.

**Methods:**

A systematic literature review across PubMed, Cochrane database, Embase, Google Scholar, and Ovid was performed according to PRISMA guidelines to identify relevant articles that discussed risk factors for cardiovascular manifestations, cardiac manifestations in COVID‐19 patients, and cardiac‐specific biomarkers with their clinical implications on COVID‐19.

**Results:**

Sixty‐one relevant articles were identified which described risk factors for cardiovascular manifestations, cardiac manifestations (including heart failure, cardiogenic shock, arrhythmia, and myocarditis among others) and cardiac‐specific biomarkers (including CK‐MB, CK, myoglobin, troponin, and NT‐proBNP). Cardiovascular risk factors can play a crucial role in identifying patients vulnerable to developing cardiovascular manifestations of COVID‐19 and thus help to save lives. A wide array of cardiac manifestations is associated with the interaction between COVID‐19 and the cardiovascular system. Cardiac‐specific biomarkers provide a useful prognostic tool in helping identify patients with the severe disease early and allowing for escalation of treatment in a timely fashion.

**Conclusion:**

COVID‐19 is an evolving pandemic with predominate respiratory manifestations, however, due to the interaction with the cardiovascular system; cardiac manifestations/complications feature heavily in this disease, with cardiac biomarkers providing important prognostic information.

## 1 INTRODUCTION

Coronavirus disease‐2019 (COVID‐19), caused by the novel severe acute respiratory syndrome coronavirus 2 (SARS‐CoV‐2), first emerged in Wuhan, China in December 2019 in patients presenting with pneumonia of unknown origin.^1^ Exponential rises in global cases and mortality, has precipitously led to a global public health crisis and subsequently declared a global pandemic by the World Health Organization (WHO) on the 21st of March 2020. With nearly 5.5 million confirmed cases and nearly 350 000 deaths, it remains a rapidly evolving situation; however, the lack of widespread testing may mean that the incidence may be higher than that reported.

Significant concerns relating to COVID‐19 and the cardiovascular system have been highlighted, with COVID‐19 inducing multiple cytokines and chemokines resulting in vascular inflammation, plaque instability, and myocardial inflammation.^1^ Additionally, pre‐existing cardiovascular disease (CVD) predisposes to COVID‐19 infection with elevated risk of adverse outcomes.^2^, ^3^ These concerns are compounded by results from previous epidemiologic and clinical studies, demonstrating that patients with pre‐existing coronary artery disease and/or risk factors for atherosclerotic disease are at an increased risk of developing acute coronary syndromes (ACS) during acute infection.^4^, ^5^, ^6^

Although COVID‐19 primarily presents with respiratory‐related symptoms, due to the interplay between COVID‐19 and the cardiovascular system, studies show a high prevalence of cardiovascular comorbidities in hospitalized patients.^7^ Cardiac manifestations have been described and we aim to systematically review the current published literature in this regards.

## 2 METHODS AND MATERIALS

### 2.1 Search strategy

A comprehensive literature search was performed on PubMed, Cochrane database, Embase, Google Scholar, and Ovid identifying articles that discussed cardiac manifestations in COVID‐19 patients and cardiac biomarkers with their clinical implications on COVID‐19 in accordance with Preferred Reporting Items for Systematic Reviews and Meta‐analysis (PRISMA) guidelines.^8^

Keywords used included “COVID‐19,” “severe acute respiratory syndrome coronavirus 2,” “SARS‐CoV‐2,” “novel human coronavirus,” “heart failure,” “cardiogenic shock,” “myocarditis,” “pericarditis,” “acute coronary syndrome,” “ACS,” “ST‐segment elevation,” “ST segment changes,” “coronary arteries,” “arrhythmia,” “risk factors,” “cardiac,” “cardiac enzymes,” “cardiac biomarkers,” “troponin,” “myoglobin,” “creatine kinase,” “CK‐MB,” “N‐terminal prohormone of brain natriuretic peptide” and “NT‐proBNP.” The search terms were used as keywords and in combination as MeSH terms to maximize the output from literature findings.

A staged literature search was performed, and relevant articles are cited and referenced within each section separately. All identified articles reference lists were analyzed for additional studies. All relevant articles were identified and screened; the results are summarized in a narrative manner in each relevant section within the text of this review, with a summary table of each section provided where appropriate.

### 2.2 Inclusion and exclusion criteria

Studies were included if they had discussed a cardiac manifestation associated with COVID‐19, correlation between cardiac‐specific biomarkers and the diagnosis or prediction of severity of COVID‐19 infection. Exclusion criteria were editorials, consensus documents, commentaries, and studies with no particular definition of the role of cardiac biomarkers in COVID‐19.

### 2.3 Data extraction

All articles were screened by two authors (AMAS, SAS) and any disagreement was reached by consensus or involvement of a third author (AH). Data were extracted by two authors (MMS, SI) and validated by a third author (AMAS).

### 2.4 Quality assessment

The quality of each publication was evaluated using the Newcastle‐Ottawa scale (Table 1). This review addressed key domains: risk factors for cardiovascular manifestation, cardiac manifestations, cardiac‐specific biomarkers, correlation with severity of CVD, survival, and outcomes.

**Table 1:** Newcastle‐Ottawa scale table

					Comparability Reporting of Cardiovascular manifestations = * Reporting of Cardiac Biomarkers = *			
	Selection					Outcomes		
Author	Representation of patients with COVID‐19	Selection of patients with Cardiovascular Manifestations	Ascertainment of exposure	Demonstration that outcome of interest was not present at start of study		Assessment of outcomes †	Follow‐up long enough for outcomes to occur	Adequacy reporting of outcomes
Chen et al^9^	*	*	*	*	*	*	*	*
Yang et al^10^	*	*	*	**‐**	*	*	*	*
Ruan et al^11^	*	*	*	*	**	*	*	*
Wang et al^7^	*	*	*	*	**	*	*	*
Shi et al^13^	*	*	*	*	**	*	*	**‐**
Zhou et al^14^	*	*	*	*	**	*	‐	*
Guo et al^15^	*	*	‐	*	**	*	*	*
Grasselli et al^16^	*	*	*	*	‐	*	*	*
Guan et al^63^	*	*	*	*	*	*	*	*
Wu et al^64^	*	*	*	*	*	*	*	*
Ruan et al^19^	*	*	*	*	*	*	*	*
Mehra et al^17^	*	*	*	*	*	*	*	‐
Aggarwal et al^18^	*	*	*	*	*	*	*	*
Du et al^20^	*	*	*	**‐**	*	*	*	*
Shi et al^12^	*	*	*	*	**	*	*	**‐**
Belhadjer et al^65^	*	*	*	‐	*	*	*	*
Ullah et al^66^	*	*	*	‐	*	*	*	‐
Fried et al^22^	*	*	*	*	*	*	‐	‐
Tavazzi et al^23^	*	*	*	‐	*	*	*	*
Sanchez‐Recalde et al^24^	*	*	*	‐	*	*	*	‐
Bemtgen et al^25^	*	*	*	*	*	*	‐	‐
Harari et al^26^	*	*	*	‐	*	*	*	*
Liu et al^27^	*	*	*	*	*	*	*	*
Shamshirian et al^67^	*	*		*	**	*	*	*
Zhang et al^28^	*	**‐**	*	*	*	*	*	*
Seecheran et al^30^	*	*	*	*	*	*	*	*
Hou et al^29^	*	*	*	*	*	*	*	*
Hui H et al^32^	*	*	‐	*	*	*	*	*
Du et al^33^	*	*	*	*	*	*	*	*
Borba et al^61^	*	*	*	*	*	‐	‐	‐
Deng et al^40^	*	*	**‐**	*	*	*	**‐**	**‐**
Zeng et al^41^	**‐**	**‐**	*	*	*	*	**‐**	*
Doyen et al^68^	**‐**	**‐**	*	*	*	*	**‐**	*
Kim et al^42^	‐	‐	*	*	*	*	‐	*
Sala et al^69^	‐	‐	*	*	*	*	‐	*
Craver et al^70^	‐	‐	*	*	*	*	‐	*
Incardia et al^71^	‐	‐	*	*	*	*	‐	*
Luetkens et al^72^	‐	‐	*	*	*	*	‐	*
Coyle et al^43^	‐	‐	*	*	*	*	‐	*
Oberweis et al^73^	**‐**	**‐**	*	*	*	*	**‐**	*
Hua et al^44^	**‐**	**‐**	*	*	*	*	**‐**	*
Courand et al^48^	**‐**	**‐**	*	*	*	*	**‐**	*
Kumar et al^49^	‐	‐	*	*	*	*	‐	*
Gasso et al^74^	‐	‐	*	*	*	*	‐	*
Bangalore et al^46^	*	*	*	*	*	*	*	*
Asif et al^47^	‐	‐	*	*	*	*	‐	*
Dominiguez‐Erquicia et al^45^	‐	‐	*	*	*	*	‐	*
Han et al^75^	*	*	*	*	*	*	*	*
Zhao et al^76^	*	*	*	*	*	*	*	*
Zheng et al^77^	*	*	*	*	*	*	*	*
Gao et al^78^	*	*	*	*	*	*	*	*
Yan et al^79^	*	*	*	*	*	*	*	*
Inciardi et al^80^	*	*	*	*	*	*	*	*
Wan et al^52^	*	*	*	*	*	*	*	*
Huang et al^1^	*	*	*	*	*	*	*	*
Yang et al^81^	*	*	*	*	*	*	*	*
Chen et al^82^	*	*	*	*	*	*	*	*
Arentz et al^83^	*	*	*	*	*	*	*	*
Liu et al^84^	*	*	*	*	*	*	*	*
Fan et al^85^	*	*	*	*	*	*	*	*
Wu et al^86^	*	*	*	*	*	*	*	*

Abbreviations: COVID‐19 ‐ Coronavirus disease 2019.

### 2.5 Statistical analysis

It was not possible to conduct an appropriate meta‐analysis due to limited research data among the studies on this subject.

## 3 RESULTS

**Figure 1:**
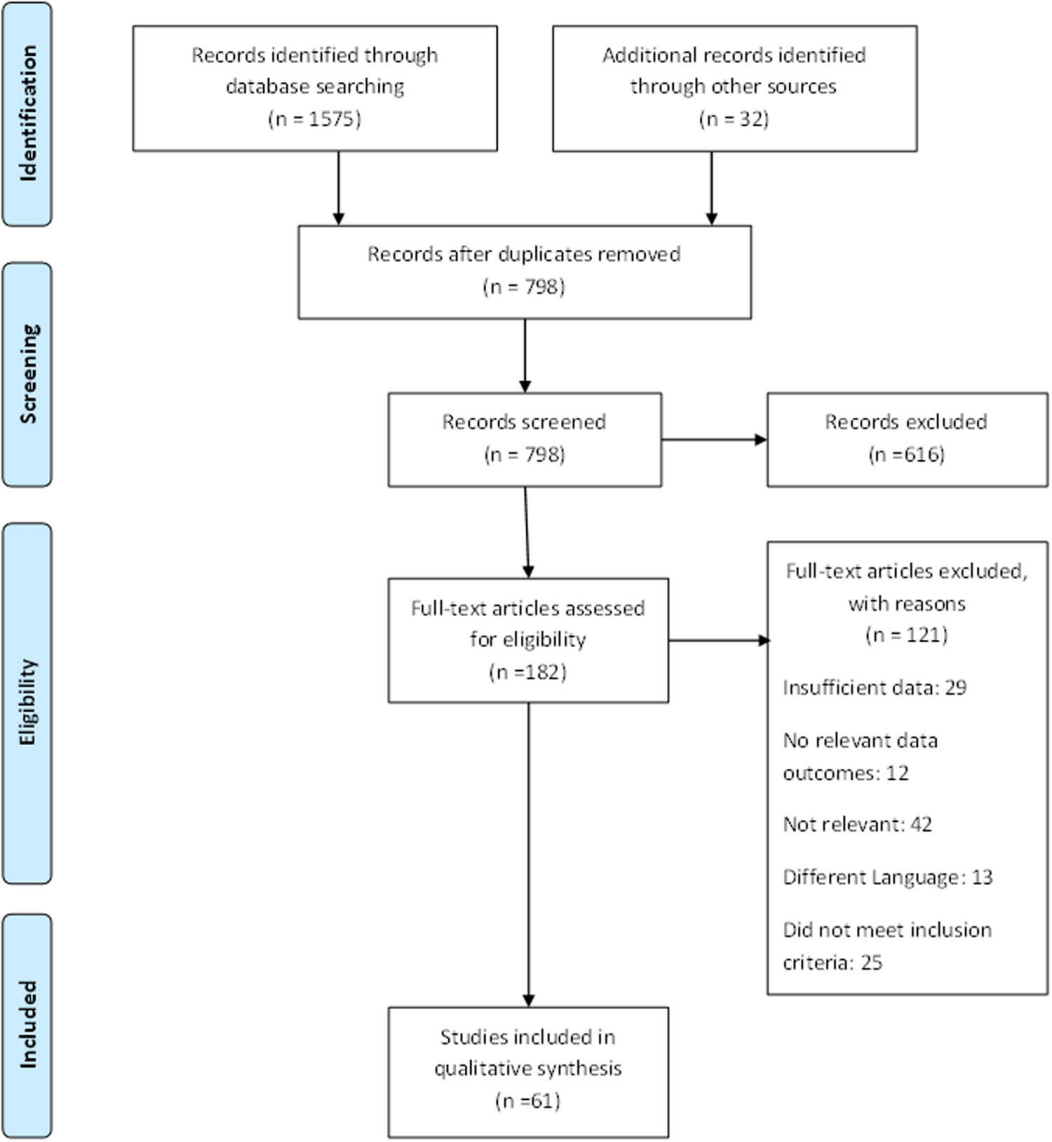
PRISMA flow diagram

A total of 1602 articles were found. Following the removal of duplicates, 798 articles were screened. Of these, 616 articles were excluded after applying the inclusion and exclusion criteria. The remaining 182 articles were analyzed in full, of which 61 articles met our inclusion criteria and were included in our analysis. The complete PRISMA flow chart is reported in Figure 1. Results are divided into risk factors for cardiovascular manifestations, cardiac manifestations, and cardiac‐specific biomarkers.

### 3.1 Risk factors for cardiovascular manifestations

Several risk factors for cardiovascular manifestation in COVID‐19 patients have been described and summarized in Table 2. One of the most important risk factors is the presence of pre‐existing cardiovascular comorbidities. Patients with hypertension or any other cardiovascular comorbidity were more likely to develop a cardiovascular complication due to SARS‐CoV‐2 infection, with a higher proportion of hypertensive patients developing acute heart injury and heart failure.^9^

**Table 2:** Risk factors for cardiovascular manifestations

Authors/country	Study type/cohort size	Patient characteristics	Cardiovascular co‐morbidities	Cardiac complications	Complication association with outcomes
Chen et al^9^ China	Retrospective 274	Median age: 62 Males: 171 (62%)	Hypertension: 93 (34%) Diabetes: 47 (17%) CVD: 23 (8%) Heart Failure: 1 (<1%) CVA: 4 (1%)	Acute cardiac injury: 89/203 (44%) Heart failure: 43/176 (24%) Shock: 46/274 (17%)	Higher levels of cardiac troponin, BNP, acute cardiac injury and cardiovascular comorbidities were recorded in deceased patients
Yang et al^10^ China	Single‐center, retrospective, observational study 52	Mean age: 59.7 ± 13.3 Males: 35 (67%)	Diabetes: 9 (17%) CVD: 5 (10%) CVA: 7 (13.5%)	Acute cardiac injury: 12 (23%)	Higher prevalence of cardiovascular co‐morbidities in non‐survivors
Ruan et al^11^ China	150	Median age: ‐ Males: 102 (68%)	Hypertension: 52 (34.7%) Diabetes: 25 (16.7%) CVD: 13 (8.6%) CVA: 12 (8%)	5 patients with myocardial damage died of circulatory failure; 22 died of respiratory failure +myocardial damage	A trend of high cardiac troponins, acute cardiac injury and cardiovascular co‐morbidities was observed in deceased patients
Wang et al^7^ China	Single‐center case series 138	Median age: 56 Males: 75 (54.3%)	Hypertension: 43 (31.2%) Diabetes: 14 (10.1%) CVD: 20 (14.5%) CVA: 7	Arrhythmia: 16 (11.6%) Shock: 11 (7.9%) Acute cardiac injury: 10 (7.2%)	Increase in ICU admissions in patients with cardiovascular co‐morbidities
Shi et al^13^ China	Descriptive 416	Median age: 64 Males:195 (46.8%)	Hypertension: 127 (30.5%) Diabetes: 60 (14.4%) CVA: 22 (5.3%) CAD: 44 (10.6%)	Acute cardiac injury: 82 (19.7%) Heart failure: 17 (4.1%)	High risk of mortality in patients with acute cardiac injury
Zhou et al^14^ China	Retrospective cohort 191	Median age: 56 Males: 119 (62%)	Hypertension: 58 (30%) Diabetes: 36 (19%) CAD: 15 (8%)	Acute cardiac injury: 33 (17%)	Occurrence of acute cardiac injury and cardiovascular comorbidities was higher in non‐survivors.
Guo et al^15^ China	Retrospective single‐center 187	Mean age: 58.5 ± 14.6 Males: 91 (48.7%)	Hypertension: 61 (32.6%) Diabetes: 28 (15%) CVD: 21 (11.2%) Cardiomyopathy: 8 (4.3%)	Myocardial injury: 52 (27.8%) Arrhythmias: VT/VF: 11 (5.8%)	High risk of mortality in patients with higher cardiac troponins.
Grasselli et al^16^ Italy	Retrospective case series 1591	Median age: 63 Males: 1304 (82%)	Hypertension: 509 (49%) Diabetes: 180 (17%) CVD: 223 (21%) Hypercholesterolemia: 188 (18%)		High mortality rates were observed in patients with hypertension
Guan et al^63^ China	1099	Median age: 47 Males: 637 (58.1%)	Hypertension: 165 (15%) Diabetes: 81 (7.3%) CAD: 27 (2.5%) CVA: 15 (1.4%)		Patients with severe COVID‐19 had higher prevalence of cardiovascular co‐morbidities
Wu et al^64^ China	Retrospective cohort 201	Median age: 51 Males: 128 (63.7%)	Hypertension: 39 (19.4%) Diabetes: 22 (10.9%) CVD: 8 (4%)		Patients with acute respiratory distress syndrome had higher frequency of hypertension and diabetes

In a study by Yang et al^10^ patients with diabetes, the cerebrovascular, and cardiovascular disease had inferior outcomes with an acute cardiac injury one of the common complications, leading to death. An analysis of 150 patients with COVID‐19 demonstrated that hypertension, diabetes, pre‐existing CVD, and cerebrovascular disease were responsible for 43%, 18%, 19%, and 10% of all deaths respectively. Of these deaths, 39% were attributed to heart failure or respiratory failure.^11^

Other studies have linked hypertension and diabetes as significant risk factors for developing cardiovascular complications.^13^, ^14^ Guo et al^15^ demonstrated the importance of troponin levels in informing the likelihood of cardiovascular manifestations occurring. Patients with elevated troponin levels had more frequent malignant arrhythmias and higher levels of other cardiac biomarkers.

An Italian retrospective study showed hypercholesterolemia was another significant comorbidity that can predict worse outcomes involving cardiovascular implications. One hundred eighty‐eight patients present in Intensive Care Units across hospitals in Lombardy had hypercholesterolemia, indicating the importance as a risk factor.^16^

The commonest risk factor associated with an adverse cardiovascular complication in COVID‐19 patients was hypertension (27%) comorbidity which contributed towards cardiac manifestations. CVD and diabetes was seen in 12.1% and 11.5% of patients, respectively.

### 3.2 Cardiac manifestations

#### 3.2.1 Heart failure and cardiogenic shock

Heart failure as a complication of pneumonia is common in hospital patients. However, several studies have identified heart failure as a significant manifestation of COVID‐19.^14^, ^17^, ^18^ One of the first studies linking heart failure and COVID‐19 involved 191 patients with COVID‐19, 44 of these patients developed heart failure with mortality rate of 64%. More than half of the deaths reported heart failure as a predicting factor.^14^

In a similar study by Ruan et al^19^ of 68 fatal cases of COVID‐19, 58% of patients died from respiratory failure, 7% from heart failure and 33% died from both. A further study concluded that heart failure was a significant complication in the latter stages of the disease, and a prominent manifestation in individuals with and without pre‐existing cardiovascular abnormalities.^9^

A multinational observational study showcased that COVID‐19 complicated with heart failure was associated with an increase risk of in‐hospital mortality.^17^ Mortality in heart failure patients was 15.3% compared to 5.6% in patients without heart failure (odds ratio, 2.48; 95% CI, 1.62‐3.79).

Reports from the US showed that congestive heart failure was important comorbidity in patients who died as a result of coronavirus as well as a new‐onset manifestation of the disease.^18^ An observational study in China of 109 deceased patients underlined the importance of acting swiftly in a hospitalized coronavirus patient, as multiple organ failure, including heart failure, could happen rapidly in a COVID‐19 patient admitted to the hospital.^20^

Heart failure is postulated to occur in COVID‐19 patients due to the severe immune system over‐reaction resulting in cytokine storm. The virus downregulates the angiotensin‐converting enzyme 2 (ACE2), leading to increased levels of Angiotensin II causing increased inflammation, hypertension, and thrombosis.^21^ As further data becomes available, the mechanism of heart failure in COVID‐19 patients will become clearer and help provide a standard approach to its treatment.

There are several case reports of COVID‐19 patients degenerating into cardiogenic shock. In a series of case reports that depicted the various cardiovascular presentations of COVID‐19, three out of four cases developed cardiogenic shock. The hemodynamic assessment was integral to the recognition of cardiogenic shock in these cases. The authors recommended that a lower threshold to assess for shock in acute systolic heart failure linked with COVID‐19 was crucial.^22^

Tavazzi et al^23^ reported localization of the SARS‐CoV‐2 in the myocardium leading to cardiogenic shock. Case reports from Spain informed that out of four patients that developed cardiogenic shock three died mortality of 75%. Notably, three of these patients had no cardiovascular risk factors or significant comorbidities.^24^ Further reports from Germany and the USA have also mentioned cardiogenic shock as a significant complication of COVID‐19.^25^, ^26^ Based on all studies, approximately 8% of patients developed heart failure/cardiogenic shock as a manifestation of COVID‐19. Table 3 summarizes the studies used in analyzing the occurrence of heart failure and cardiogenic shock in COVID‐19 patients.

**Table 3:** Heart failure and cardiogenic shock

Author	Study design	Country	Cohort size	Comments
Zhou et al^14^	Retrospective cohort study; multicentre	China	191	44 (23%) patients developed heart failure as a complication of COVID‐19.
Ruan et al^19^	Retrospective cohort study; multicentre	China	150	27 (40%) of the 68 fatal patients had heart failure as a cause of death.
Chen et al^9^	Retrospective cohort study; single center	China	799	Heart failure was a major complication in deceased patients with or without pre‐existing comorbidities.
Mehra et al^17^	Multinational observational study	Europe, North America, Asia	8910 (169 hospitals)	Heart failure was associated with an increased risk of in‐hospital death in COVID‐19 patients.
Aggarwal et al^18^	Retrospective cohort study; single center	USA	42	13% of patients developed heart failure as a complication of SARS‐CoV‐2 infection.
Du et al^20^	Multicentre observational study	China	109 decedents	Multiple organ failure, especially heart failure and respiratory failure occurred rapidly after hospital admission in all patients.
Shi et al^12^	Retrospective cohort study; Single center	China	671	Acute heart failure was the cause of death of 19.4% of the deceased patients.
Belhadjer et al^65^	Retrospective cohort study; Multicentre	France and Switzerland	35	One‐third of all the children developed acute heart failure associated with COVID‐19 and multisystem inflammatory syndrome
Ullah et al^66^	Case Report	USA	1	COVID‐19 patient developed acute pulmonary embolism and right‐sided heart failure.
Fried et al^22^	Case Reports	USA	4	Three out of four cases reported cardiogenic shock as a manifestation of COVID‐19.
Tavazzi et al^23^	Case Report	Italy	1	Myocardial localization of SARS‐CoV‐2 led to patient degenerating into respiratory distress, hypotension and cardiogenic shock
Sanchez‐Recalde et al^24^	Case Reports	Spain	7	4 COVID‐19 patients developed cardiogenic shock, resulting in 3 deaths (75% mortality).
Bemtgen et al^25^	Case Report	Germany	1	COVID‐19 patient presenting with acute respiratory distress syndrome degenerates into cardiogenic and vasoplegic shock.
Harari et al^26^	Case Report	USA	1	Patient developed acute myocardial infarction complicated by coronary thrombosis and cardiogenic shock.

#### 3.2.2 Arrhythmias

Patients affected with COVID‐19 are at an increased risk of arrhythmias due to underlying comorbidities, polypharmacy, and disease progression. In a study of 137 patients, 7.3% reported palpitations as one of their symptoms.^27^ Several studies have concluded that the prevalence of cardiac arrhythmias is higher in critically ill patients compared to non‐critically ill patients.^7^, ^28^, ^29^ Although in most studies the specific cause or types of arrhythmias were not recorded, arrhythmias occurred in around 14% of patients affected with COVID‐19. A general trend of tachyarrhythmias was observed overall in patients with COVID‐19. With atrial fibrillation (7%), atrial flutter, and ventricular tachycardia/ventricular fibrillation (5.9%) being the likely pathologic arrhythmias.

The association of underlying CVD and myocardial injury with fatal outcomes in COVID‐19 patients has been highlighted by a retrospective study of 187 patients. Among the patients studied, 11 (5.9%) reported ventricular tachycardia/ventricular fibrillation. Patients with higher cardiac troponin (Tn) levels had a significantly higher incidence of malignant arrhythmias than those with normal Tn levels (11.5% vs 5.2%; *P* < .001).^15^

A case report by Seecheran et al reported findings of a Caribbean‐Black male who presented with tachycardia and atrial flutter with 2‐to‐1 atrioventricular block. This later transitioned into atrial fibrillation with rapid ventricular response. The patient also illustrated electrolyte abnormalities such as hypokalaemia and hypomagnesemia.^30^ Additionally, the risk of arrhythmia is likely to increase with the development of infection, and as the severity and/or systemic inflammatory response increases. Studies investigating the same revealed that cardiac arrhythmias were associated with higher in‐hospital mortality.^31^

Furthermore, in a study of 41 patients, Hui et al^32^ documented the heart rate of 17 patients, three of whom presented with tachycardia. Atrial fibrillation was also reported in two patients with critical illness both of whom had a fatal outcome. Similarly, Du et al^33^ analyzed 85 fatal cases of COVID‐19 and concluded that some form of arrhythmia was present in 60%, with cardiac arrest or malignant arrhythmia being the cause of death for over 10% of cases.

Of note, non‐specialized clinicians may use multiple concurrent medications that could potentially (synergistically, in some cases) contribute to an increased arrhythmic risk. Several trials are underway testing combination therapies, for instance, a Brazilian study compared low vs high dose chloroquine, in combination with ceftriaxone and azithromycin with or without oseltamivir.^33^ This study amongst others was terminated due to safety concerns as 25% of patients in the high‐dose arm showed QT prolongation and two experienced ventricular tachycardia before death. Table 4 summarizes the studies used in analyzing the occurrence of arrhythmias in COVID‐19 patients.

**Table 4:** Arrhythmias

Author	Study design	Country	Sample size	Patient characteristics	Occurrence	Comments
Liu et al^27^	Retrospective, single‐center	China	137	Median age: 57 y Males: 61 (44.5%)	10 patients reported palpitations	Heart palpitations were considered a low frequency occurrence according to this patient population
Wang et al^7^	Retrospective, single‐center case series	China	138	Median age: 56 y Males: 75 (54.3)	23 patients (16.7%) *P* value: <.001	Arrhythmia was one of the common complications in this patient group
Shamshirian et al^67^	Meta‐analysis	Iran	3473 (16 papers)	…	11% (odds ratio: 22.17)	Arrhythmias are significantly associated with ICU admissions in COVID‐19 patients
Zhang et al^28^	Retrospective case series; single center	China	221	Median age: 55 yMales: 48.9%	22 patients (10.9%) *P* value <.001	Arrhythmia was a common complication in this patient group. Compared to non‐severe patients, occurrence of arrhythmia in severe patients was significantly high.
Seecheran et al^30^	Case report	Trinidad		Age: 46 Male	Tachycardia; patient developed atrial fibrillation with rapid ventricular response; electrolyte abnormalities were observed	The patient experienced atrial arrhythmias (AFL, AF) which resolved with rate and rhythm control strategies, and supportive care.
Guo et al^15^	Case series study	China	187	Mean age: 58.5 ± 14.6 males: 91 (48.7%)	11 patients (5.8%) *P* value: <.001	Patients with elevated cardiac troponins had a higher frequency of arrhythmias.
Hou et al^29^	Retrospective Cohort Study	China	101	Median age: 50.9 ± 20.1 y Males: 44 (43.6%)	7 patients (*P* value: <.001)	Higher incidence of arrhythmias in the disease progression group.
Hui H et al^32^	Retrospective, single‐center	China	41	Median age: 47 y Males: 19 (46.3)	3 patients (6.4%)	Results suggest that main attention should be paid on monitoring the high‐risk factors of arrhythmia and cardiac function.
Du et al^33^	Retrospective; observational study	China	85	Median age: 65.8 y Males: 62 (72.9%)	51 patients (60%)	The study concluded arrhythmia to be a common complication. Additionally, malignant arrhythmias were a common cause of death.
Borba et al^61^	Randomized control trial	Brazil	81	Median age: 51.1 y Males: 61 (75.3%)	25% patients showed QT prolongation; two experienced ventricular tachycardia	The trial was terminated due to safety concerns.

#### 3.2.3 Cardiac inflammatory and coronary manifestations of COVID‐19

Myocardial injury in COVID‐19 is a recognized phenomenon. Case series include reports of myocarditis, ACS, and spontaneous coronary artery dissection (SCAD) (Table 5). Myocarditis was reported with an incidence of 12.5% in one cohort study, ACS was noted in 33% of patients presenting with ST elevation in a case series and SCAD and have been reported in three patients to date.

**Table 5:** Cardiac inflammatory and coronary manifestations of COVID‐19

Author	Study design	Country	Sample size	Cardiac manifestation	Comments
Deng et al^40^	Retrospective cohort	Wuhan, China	112	Myocarditis	14/112 (12.5%) presented with abnormalities similar to myocarditis, but this was unconfirmed by ECG/echocardiogram.
Zeng et al^41^	Case report	China	1	Myocarditis	First known case of a COVID positive patient presenting with myocardial injury. The patient presented with features of COVID‐19 pneumonia and satisfied Chinese consensus statement to be diagnosed with myocarditis (due to high troponins and myocardial dyskinesia). Troponin and LVEF improved following antiviral therapy with liponavir‐ritonavir, immunoglobulin, interferon, and methylprednisolone.
Doyen et al^68^	Case report	Italy	1	Myocarditis, changes consistent with acute coronary syndrome	63 y old male patient developed adult respiratory distress syndrome secondary to COVID 19. During ITU stay, changes consistent with Non‐ST segment elevation myocardial infarction were noted. Coronary angiography showed no disease and cardiac MRI showed subendocardial enhancement consistent with myocarditis. The patient was treated with hydrocortisone.
Kim et al^42^	Case report	South Korea	1	Myocarditis	21 y old female presented with symptoms consistent with COVID‐19 pneumonia. Cardiac CT showed myocardial edema and subendocardial perfusion defects. Myocarditis was confirmed with multimodality imaging.
Sala et al^69^	Case report	Italy	1	Myocarditis	43 y old female presented with chest pain and dyspnea and she tested positive for COVID 19. Coronary CT angiography showed hypokinesia of the left ventricle and basal segments with normal apical segments (reverse Takotsubo cardiomyopathy). Endocardial biopsy was consistent with myocarditis with diffuse T cell lymphocytic infiltrate. The patient was treated with liponovir/ritonavir and hydroxychloroquine. She was discharged on day 13 after presentation.
Craver et al^70^	Case report	USA	1	Myocarditis	Previously healthy 17 y old died following several days history of headaches, dizziness, and vomiting and was found following an out of hospital cardiac arrest. He was confirmed COVID‐positive with post‐mortem swabs. Autopsy findings revealed prominent eosinophilic infiltrates in myocardial tissue.
Incardia et al^71^	Case report	Italy	1	Myocarditis	53 y old female presented with the influenza‐like symptoms of COVID‐19 before developing heart failure. ECG showed diffuse ST elevation, with elevated troponins and BNP. The diagnosis was confirmed using cardiac MRI. The patient was treated with liponavir/ritonavir, chloroquine, steroids, and heart failure medication.
Luetkens et al^72^	Case report	Germany	1	Myocarditis	79 y old male presented with dyspnea, fatigue, and recurrent syncope. He had radiological findings of COVID 19 pneumonia, accompanied by pleural effusions. Multiparametric cardiac MRI showed diffuse myocardial edema and infiltration consistent with myocarditis.
Coyle et al^43^	Case report	USA	1	Myocarditis	57 y old male presented with symptoms of COVID 19 pneumonia and subsequently developed ARDS. Troponin and BNP were elevated with no ST changes. Echocardiogram showed diffuse hypokinesis and reduced ejection fraction. Cardiac MRI showed diffuse edema. The patient was treated with hydroxychloroquine, azithromycin, ceftriaxone, methylprednisolone and tocilizumab.
Oberweis et al^73^	Case report	Belgium	1	Myocarditis	8 y old male presented with fever, coughing, weight loss and severe fatigue. He had lymphopenia, raised CRP, troponins, d‐dimers, and IL6. ECG showed discrete ST elevation consistent with pericarditis, with MRI showing evidence of diffuse myocardial edema. He was admitted to PICU and treated with IV immunoglobulins and dobutamine.
Hua et al^44^	Case report	USA	1	Cardiac tamponade, ST changes	47 y old male presented with chest pain and breathlessness, subsequently tested positive for COVID 19. Echocardiogram showed cardiac tamponade with ST elevation in infero‐lateral leads.
Courand et al^48^	Case report	France	1	Coronary artery dissection	53 y old male presented with symptoms consistent with COVID‐19 pneumonia and was confirmed positive on PCR. Angiography was performed due to elevated troponins and ECG changes of T wave inversions and showed a spontaneous dissection in the mid‐right coronary artery. The patient was managed conservatively with aspirin.
Kumar et al^49^	Case report	USA	1	Coronary artery dissection	48 y old female with cardiovascular risk factors presented with retrosternal chest pain. She had no ischemic changes on ECG. She was found to have a dissection of the mid‐to‐distal left anterior descending (LAD) artery. Following discharge with medical therapy, she re‐presented with chest pain, ST elevation in infero‐lateral leads and raised troponins. Found to be COVID‐19 positive and angiogram showed ostium of LAD to the distal vessel.
Gasso et al^74^	Case report	Spain	1	Coronary artery dissection	39 y old male presented with symptoms of COVID 19 and required ventilatory support. During his stay, he developed ST segment changes in inferior leads but he remained asymptomatic. Angiography showed spontaneous coronary artery dissection (SCAD) of the first obtuse marginal branch. Autoimmune and rheumatologically causes were ruled out.
Bangalore et al^46^	Case series	USA	18	ST‐segment elevation	18 COVID‐positive patients from 6 hospitals in New York were studied. 83% were men with 33% having chest pain at the time of presentation. 56% presented with ST elevation while the rest developed it during hospitalization. There was a high prevalence of non‐obstructive disease and 72% died in hospital.
Asif et al^47^	Case report	USA	2	ST‐segment elevation	A 63 y old male and 71 y old female tested positive for COVID‐19, developed ARDS, and required ventilatory support. During their stay, they developed ST elevation and raised troponins. They were treated medically for acute coronary syndrome, followed by resolution of ECG and reduction in troponins.
Dominiguez‐Erquicia et al^45^	Case report	Spain	1	Coronary artery thrombosis, acute coronary syndrome	64 y old male presented with ST‐elevation MI, a day after being discharged from hospital following treatment for COVID‐19 pneumonia. He had no pre‐existing cardiovascular risk factors. Angiography showed critical stenosis of proximal right coronary artery stenosis and a filling defect in the left anterior descending artery. He required coronary artery stenting.

Myocarditis, defined as myocardial injury and inflammation without an ischemic cause, can be attributed to infection (predominantly viral), rheumatological disease, and malignancy. The proposed pathophysiology of viral myocarditis is based on activation of interleukin‐6 (IL‐6) and triggering of a subsequent cytokine storm, combined with direct myocardial injury.^34^ This feature is consistent with the observed augmentation of inflammatory markers seen in case reports detailing myocarditis in COVID‐19. Additionally, human coronaviruses including Sars‐Cov‐1 and MERS‐Cov have been isolated in mammalian cardiac tissue, indicating the possibility of direct toxic effects on the heart.^35^, ^36^ Sars‐Cov‐2 enters cells by attaching to the ACE‐2 receptor, which is present in the heart and upregulated in heart failure, suggesting a mechanism for direct effect of the virus on cardiac tissue.^37^ Diagnosis of myocarditis can be done with various techniques, which could impact upon its perceived prevalence within the population. The American Heart Association recommends echocardiography or cardiac magnetic resonance imaging (MRI), with definitive diagnosis requiring an endomyocardial biopsy. In the absence of a cardiac MRI, contrast‐enhanced CT is recommended.^38^

The first cohort of patients with COVID‐19 and myocarditis was reported from China.^39^ A retrospective observational study set the prevalence at 12.5% (14/112), however, the diagnosis was made using consensus criteria, and accompanying ECG or echocardiogram changes were not seen. It has been reported in at least six patients around the world, ranging in age from 8 to 79 years (Table 4). Subsequent case reports confirmed diagnosis using cardiac MRI with gadolinium washout, combined with echocardiogram findings.^40^, ^41^, ^42^ Treatment was variable, liponovir/ritonavir and steroids were the most common combination, with one case reporting the use of tocilizumab (anti‐IL6).^43^

ACS is a recognized complication of COVID‐19, its pathophysiology may be related to the hypercoagulable state induced by the virus, causing thrombosis of coronary arteries.^44^ A case report from Spain detailed the presentation of a 64‐year‐old male with ACS following discharge from hospital due to COVID‐19 who possessed no cardiovascular risk factors, and underwent PCI.^45^ In a case series of 18 patients, it was noted that only 33% (6/18) of patients with ST‐elevation had chest pain and 72% died in hospital.^46^ Asif et al details the cases of two patients admitted to ITU with ARDS caused by COVID‐19, who subsequently developed ST elevation and elevated troponins. They were treated conservatively and the changes were resolved during their stay.^47^

Spontaneous coronary artery dissection (SCAD) has been reported at least 3 times in the literature, in patients with varying ages and risk factors in association with COVID‐19. Courand et al detailed the case of a 53‐year‐old female who presented with symptoms of COVID‐19 and angiography was performed due to elevated troponins and ST changes. She was found to have SCAD of the right coronary artery and due to the absence of symptoms she was treated conservatively with aspirin.^48^ In contrast, Kumar et al^49^ reported the history of a 48‐year‐old female who presented with retrosternal chest pain but did not have ischemic changes reflected on her ECG on initial admission. After she was discharged, she re‐presented with infero‐lateral ST elevation and troponin elevation. Angiography confirmed SCAD and she was positive for COVID‐19, despite not presenting with symptoms consistent with pneumonia.

#### 3.2.4 Cardiac‐specific biomarkers

Cardiac biomarkers are important in recognizing patients that might be presenting with early signs of myocardial injury secondary to COVID‐19,^50^ as the presence of myocardial injury was associated with over 50% mortality.^14^ We summarized studies looking at cardiac‐specific biomarkers (CK, CK‐MB, Troponin, Myoglobin, and BNP) in Table 6. Studies that reported the frequency of elevated cardiac biomarkers, NT‐proBNP was elevated in 28% (106/380), Troponin 17% (278/1659), CK 18% (84/466) and CK‐MB 12% (133/1148).

**Table 6:** Cardiac‐specific biomarkers

Author/country	Study design	Sample size	Cardiac biomarker studied	Results
Han et al,^75^ China	Retrospective, single‐center study	273 (198‐mild, 60‐severe, 15‐critical) Median age in mild group 58.95, severe group 58.97, severe group 57.27	CK‐MB (0‐5 ng/mL) Myohaemoglobin (0‐110ug/L) Cardiac troponin I (ultra‐TnI) (0‐0.04 ng/mL) NT‐proBNP (0‐900 pg/mL)	CK‐MB raised in 10 patients No. of cases with raised CK‐MB showed no significant difference between mild, severe, and critical groups. MYO raised in 29 patients, Ultra‐TnI raised in 27 patients, NT‐proBNP raised in 34 patients. No. of cases with raised MYO, ultra Tn‐I and NT‐proBNP significantly higher in severe and critical cases compared to mild (*P* < .05) NT‐proBNP and MYO significantly increased in severe and critical cases compared to mild (*P* < .0167), but no difference between severe and critical cases. The increased ultra‐TnI significant between mild and severe cases only (*P* < .0167). The increased level of MYO, Ultra‐TnI, and NT‐proBNP was associated with the severity of COVID‐19. The case fatality rate was 22.81% (13/57) in the group with abnormal parameters compared to 5.09% (11/216) in the normal parameter group. All four parameters significantly higher in the death group compared to alive group (*P* < .001)
Zhao et al,^76^ China	Retrospective, single‐center study	91 (30 –severe, 61‐mild) Median age 46 (50.5 in severe and 42 in mild groups respectively, *P* = .049)	Cardiac troponin I (CTnI) Creatine kinase (CK) CK‐MB	CTnI raised in 3 patients, 2 in severe and 1 in mild group (3.3%) CK elevated in 14 patients 8 in severe and 6 in mild groups (15.4%) CK‐MB raised in 4 patients, 3 in severe and 1 in mild group (4.4%) CK raised more in severe compared to mild group (26.7% vs 9.8%, *P* = .018) Severe group tended to suffer damage to the cardiovascular system (26.7% vs 9.8%, *P* = .04)
Zheng et al,^77^ China	Retrospective study	99 (32 critical, 67 noncritical) Mean age in critical group of 63.8 and 42.5 in noncritical group (*P* < .001), Overall mean age 63.8	CKMB Myoglobin High sensitivity troponin T (TNTHSST) NT‐proBNP	CKMB raised in critical group compared to noncritical group (*P* = .053) Myoglobin raised in critical group compared to noncritical group (*P* = .026) TNTHSST raised in critical group compared to noncritical group (*P* = .000) NT‐proBNP raised in critical group compared to noncritical group (*P* = .022) Critically ill patients showed significant laboratory evidence of myocardial damage compared to noncritical group, TNTHSST (*P* < .001), CKMB, myoglobin, and NT‐proBNP (*P* < .05). Critically ill patients had increased myocardial damage and cardiac function indexes. Myoglobin >97.5 ng/mL, TNTHSST > 24.8 pg/mL, NT‐proBNP >1085.5 pg/mL were relatively dangerous and demonstrated a manifestation of critical illness. Elderly patients exhibited evidence of higher myocardial damage and higher levels of NT‐proBNP
Gao et al,^78^ China	Retrospective, single‐center study	54 Low baseline of NT‐proBNP </= 88.64 pg/mL =24 and high NT‐proBNP >0/88.64 pg/mL = 30 Mean overall age of 60.4, 51.6 in low group and 67.4 in high NT‐proBNP group	NT‐proBNP CK‐MB Myoglobin High sensitivity troponin I (hs tnI)	NT‐proBNP higher in the NT‐proBNP >0/88.64 pg/mL (*P* < .001) Myoglobin was higher in the group in the NT‐proBNP >0/88.64 pg/mL (*P* < .001) CK‐MB was higher in the NT‐proBNP >0/88.64 pg/mL (*P* < .001) Hs‐TnI was higher in the NT‐proBNP >0/88.64 pg/mL (*P* = .001) Univariate analysis showed a hazard ratio (HR) of NT‐proBNP associated to in‐hospital death was 1.369 (95% CI, 1.217‐1.541; *P* < .001) for an increase of 100 pg/mL For myoglobin per 1 ng/mL HR 1.006 (95% CI, 1.003‐1.008; *P* < .001) For CK‐MB per 1 ug/L HR 1.259 (95% CI, 1.098‐1.443; *P* = .001) For Hs‐TnI per 1 ng/mL HR 1.862 (95% CI, 1.273‐2.722; *P* = .001) Multivariate Cox proportional hazards regression to evaluate the independent prognostic effect of NT‐proBNP after adjust for Myoglobin (HR, 1.001, 0.996‐1.005, *P* = .773), CK‐MB (HR, 1.119, 0.905‐1.385, *P* = .299), Hs‐TnI (HR, 1.031, 0.574‐1.855; *P* = .918) overall HR 1.360 (1.177‐1.572; *P* < .001) Receiver operation characteristic curve to analyze prognostic value of the best cut off of NT‐proBNP for prediction of in‐hospital death, cut off of 88.64 pg/mL with a sensitivity of 100% and specificity of 66.67% for in‐hospital mortality. NT‐proBNP was positively correlated with cardiac injury markers (Myoglobin, CK‐MB, hs‐TnI) After adjusting for potential cofounders NT‐proBNP presented as an independent risk factor for in‐hospital death in patients with severe COVID‐19 infection.
Yan et al,^79^ China	Retrospective, single center study	193 (48 had diabetes, 145 nondiabetic) median age of 64	CK (normal </= 170 U/L) NT‐proBNP (normal <285 pg/mL) Cardiac Troponin I (normal </= 15.6 pg/mL)	On admission patients with diabetes had higher levels of NT‐proBNP 665 pg/mL vs 259 pg/mL, *P* = .007) Non‐survivors compared to survivors with diabetes had higher levels of CK (207 vs 76.5, *P* = .013), cardiac troponin I (43.1 vs 1.9, *P* < .001) and NT‐proBNP (970 vs 46, *P* < .001)
Inciardi et al,^80^ Italy	Retrospective, single‐center study	99 (two‐group, pts with cardiac disease history = 53 and noncardiac disease history = 46	High sensitivity troponin T hs TnT (normal <14 ng/L) NT‐prBNP (normal <125 pg/mL in patients 0‐74 and <450 pg/mL in patients older)	Hs TNT higher in cardiac group 34 vs 16 (*P* < .001) NT‐proBNP higher in cardiac group 2584 vs 180 (*P* < .001) NT‐proBNP and hs TNT higher at time of hospitalization in non‐survivors compared to survivors. High levels of NT‐proBNP and hs TNT were associated with poor outcomes
Wan et al,^52^ China	Case series	135 (40‐severe median age 56, 95‐mild median age 44)	CK	CK higher in severe group, 82 vs 57 (*P* = .0016) CK increased significantly in severe patients
Huang et al,^1^ China	Prospective single‐center study	41 (28 not needing OCU care and 13 needing ICU care)	Hypersensitive troponin I	5 patients had raised levels (4 in the group needing ICU care and only 1 in the group not needing ICU care, *P* = .017)
Yang et al,^81^ China	Retrospective single‐center study	92 deceased patient with COVID‐19	Cardiac troponin (cTnI) [0‐0.04 ng/mL] CK‐MB [0‐5 ng/mL] Myoglobin [0‐110ug/L]	Inpatient that developed cardiac complications, cTnI was significantly raised 2.47 vs 0.02, *P* = .016, CKMB was not significantly raised 6.82 vs 2.9, *P* = .227; Myoglobin was significantly raised 629 vs 26.3, *P* < .01
Ruan et al,^11^ China	Retrospective multicenter study	150 (82 patients discharged, 68 deaths)	Cardiac troponin [2‐28 pg/mL] Myoglobin [0‐146.9 ng/mL] CK [50‐310 U/L]	Troponin in the group that died (30.3 vs 3.5; *P* < .001), myoglobin also raised in the group that died (258.9 vs 77.7; *P* < .001) CK not significant raised in group that died (319.4 vs 231.7; *P* = .56)
Shi et al,^12^ China	Retrospective single‐center study	671 (Survivors 609, died 62)	CK‐MB [0‐5 ng/mL] Myoglobin [0‐110ug/L] cTnI [0‐0.04 ng/mL] NT‐proBNP [0‐900 pg/mL]	All biomarkers significant higher in group that died compared to survivors CK‐MB (3.6 vs 0.8, *P* < .001) Myoglobin (268 vs 32, *P* < .001) cTnI (0.235 vs 0.006, *P* < .001) NT‐proBNP (1819 vs 132, *P* < .001) Higher initial levels of CK‐MB, myoglobin, and cTnI were associated with higher mortality cTnI was significantly associated with in‐hospital morality following multivariable Cox regression analysis (HR, 1.9; CI, 1.44‐2.49)
Chen et al,^82^ China	Cross‐sectional study	150 (126 mild and 24 critical cases)	NT‐proBNP cTnI	NT‐proBNP and cTnI significantly raised in critical cases *P* < .005. Univariate logistic regression analysis showed that elevated NT‐proBNP and cTnI significantly correlated with critical disease status *P* < .05. Multivariate logistic regression analysis showed that elevated cTnI (OR = 26.909; 95%CI, 4.086‐177.226; *P* = .001) was an independent risk factors of critical disease status
Arentz et al,^83^ USA	Case series	21 (patients admitted to intensive care)	Troponin, NT‐proBNP	3 patients had a troponin higher than 0.3 ng/mL Mean NT‐proBNP was 4720 pg/mL
Shi et al,^13^ China	Retrospective single‐center study	416 (with 82 and without cardiac injury 334)	CK‐MB Myohaemoglobin hsTnI NT‐proBNP	In the group that developed cardiac injury they had significantly higher biomarkers CK‐MB (3.2 vs 0.9, *P* < 0.001), myohaemoglobin (128 vs 39; *P* < .001), hsTnI (0.19 vs <0.006, *P* < .001) NT‐proBNP (1689 vs 139, *P* < .001) The mortality rate was higher among patients with vs without cardiac injury (42 [51.2%] vs 15 [4.5%]; *P* < .001) The mortality rate increased in association with the magnitude of the reference value of hs‐TNI multivariable adjusted Cox proportional hazard regression model showed a significantly higher risk of death in patients with cardiac injury than in those without cardiac injury, either during time from symptom onset (hazard ratio [HR], 4.26 [95% CI, 1.92‐9.49]) or time from admission to study endpoint (HR, 3.41 [95% CI, 1.62‐7.16])
Zhou et al,^14^ China	Retrospective, multicenter cohort study	191 (survivors 137, non‐survivors 54)	CK U/L hs cTnI pg/mL	CK was higher in the non‐survivor group (39 vs 18; *P* = .001 hs cTnI higher in non‐survivor group (22.2 vs 3; *P* < .0001) Univariate analysis showed that a hs cTnI >28 pg/mL (OR, 80.07; CI, 10.34‐620.36; *P* < .0001) and CK > 185 U/L (OR, 2.56; CI, 1.03‐6.36; *P* = .043) were with death.
Wang et al,^7^ China	Retrospective, single‐center study	138 (ICU 36, non‐ICU 102)	CK normal range <171 U/L CK‐MB normal range <25 U/L hs cTnI normal range <26.3 pg/mL	CK higher in group needing ICU but not significant (102 vs 87, *P* = 0.08) CK‐MB significantly higher in ICU group (18 vs 13; *P* < .001) hs cTnI significant higher in ICU group (11 vs 5.1; *P* = .004)
Liu et al,^84^ China	Case series	12	CK, myoglobin, cardiac troponin I, BNP, CK‐MB	One patient was found to high levels of all biomarkers
Guo et al,^15^ China	Single‐center, retrospective, observational study	187 (135 with normal TnT level and 52 with elevated levels	TnT	Mortality was markedly higher in patients with elevated plasma TnT levels than in patients with normal TnT levels (31 [59.6%] vs 12 [8.9%]) Those with elevated TnT levels had significantly higher levels of other biomarkers of cardiac injury, specifically CK‐MB (median [IQR], 3.34 [2.11‐5.80] vs 0.81 [0.54‐1.38], ng/mL, and myoglobin (median [IQR], 128.7 [65.8‐206.9] vs 27.2 [21.0‐49.8] µg/L (*P* < .001, for all) and also had higher levels of N‐terminal pro‐brain natriuretic peptide (NT‐proBNP) (median [IQR], 817.4 (336.0‐1944.0] vs 141.4 [39.3‐303.6] pg/mL Plasma TnT levels in patients with COVID‐19 correlated significantly with both plasma high‐sensitivity C‐reactive protein levels (*β* = .530, *P* < .001) and plasma NT‐proBNP levels (*β* = .613, *P* < .001) Both TnT and NT‐proBNP levels increased significantly during the course of hospitalization in those who ultimately died, but no such dynamic changes of TnT or NT‐proBNP levels were evident in survivors.
Fan et al^85^ China	Retrospective study	101 (patients that died from COVID‐19 in the ICU)	hsTroponin I (U/L; normal range 0‐10) BNP (pg/mL; normal range 0.0‐100) CK‐MB (U/L; normal range 0‐24)	Troponin was increased I 50.5% of patients at admission to ICU and that increased to 72.28% from 48 h to death BNP was increased in 26.63 patients at admission to ICU CK‐MB was increased in 31.68% of patients at admission to ICU and increased to 55.45% of patients at 48 h to death.
Wu et al,^86^ China	Retrospective study	188	Hs TnI, CK‐MB	Patients with high levels of high‐sensitivity cardiac troponin I (hs‐TNI) on admission had significantly higher mortality (50.0%) than patients with moderate or low levels of hs‐TNI (10.0% or 9.1%). hs‐TNI level on admission was significantly negatively correlated with survival days (*r* = −.42; 95% CI = −0.64 to −0.12; *P* = .005)

Elevation in troponin is believed to reflect non‐coronary disease as evidenced by the increase in other acute phase reactants seen in critically unwell COVID‐19 patients.^51^ A retrospective cohort analysis showed that cTnI was significantly more raised in patients that died from COVID‐19 infection than those that survived (*P* < .0001).^14^ A further study showed that 19.7% of patients with COVID‐19 presented with myocardial injury diagnosed by elevated cTnI, and had a significantly higher mortality rate compared to patients with normal cTnI levels, 51.2% vs 4.5%, illustrating the potential prognostic value of cTnI.^13^

The potential prognostic value of cTnT is further exemplified in a study by Guo et al, where it was elevated in 27.8% of hospitalized patients all of which developed myocardial injury, with a mortality of 59.6% vs 8.9% in patients with normal levels. High mortality was seen in patients with elevated cTnT even without a history of CVD, 37.5%, compared to 69.4% in patients with elevated cTnT and pre‐existing CVD. Patients who had normal cTnT levels and CVD had a mortality of 13.3%.^15^ Interestingly this study showed a positive correlation between cTnT and CRP (*P* < .001) suggesting a link between the severity of systemic inflammation and myocardial injury.^15^ Elevated levels of NT‐proBNP were significantly associated with elevated cTnT levels (*P* < .001), increasing with clinical deterioration.^15^

Additionally, CK‐MB may be of prognostic value, as in a study by Wang et al, 26.1% of patients with COVID‐19 required ICU admission all of whom had significantly raised troponin and CK‐MB (*P* = .004 and *P* < 0.001 respectively),^7^ with similar results echoed in the study by Zhou et al.^14^ The study by Wan et al^52^ found that CK was also significantly raised in patients with severe disease compared to mild disease (*P* = .0016)

## 4 DISCUSSION

The presence of pre‐existing CVD and/or myocardial injury has resulted in inferior outcomes in COVID‐19 patients, in terms of mortality and morbidity. Patients with pre‐existing cardiovascular co‐morbidities are presenting with severe cases of COVID‐19 infection and represent over 20% of all fatalities with a case fatality rate reported to be 10.5%.^7^, ^53^, ^54^

Theories addressing the association between COVID‐19 and the cardiovascular system have been postulated, with COVID‐19 potentially exacerbating CV risk factors and pre‐existing CVD or potentially increasing the susceptibility of developing new CV complications.

Systemic cytokines released during COVID‐19 infection have the potential to stimulate leukocytes adhesion molecule expression on endothelial cells overlying pre‐existing atheroma, further potentiating local recruitment of these inflammatory cells. Subsequently, these changes in pre‐existing plaques can potentiate their ability to disrupt and provoke an acute coronary event.^55^, ^56^ Systemic inflammation can increase vascular shear stress at the level of the coronary arteries resulting in plaque rupture and myocardial infarction.^57^ Another explanation for the observed incidence of myocardial injury has been postulated to be due to poorly understood pro‐thrombotic inflammatory sequelae from viral infections,^58^, ^59^ with myocardial infarction well established in influenza infections at similar prevalence, suggesting that the pro‐thrombotic effects observed in COVID‐19 patients are a result of the overall inflammatory state rather than a COVID‐19 specific phenomenon. Previous studies have shown an association between influenza and myocardial infarction, myocarditis and exacerbated heart failure.^60^

The lack of large‐scale data on cardiogenic shock and COVID‐19 makes it difficult to draw any firm conclusions. However, current data informs us that cardiogenic shock is a cardiovascular manifestation that doctors must look out for in COVID‐19 patients. Finally, further studies are required to characterize the nature and classification of arrhythmias amidst the COVID‐19 pandemic.^61^

Ruan et al^11^ showed patients that died from COVID‐19 the cause of death was respiratory failure and cardiac injury in 33%, supported by Shi et al.^13^ The presence of myocardial injury was associated with a significantly worse prognosis. A meta‐analysis by Li et al cardiac biomarkers were significantly higher in severe cases compared to milder cases (*P* < .001), this included troponin (*P* < .001), CK‐MB (*P* < .001) and NT‐proBNP (*P* = .009) but myoglobin was not (*P* = .052). Additionally, death was also higher in patients with acute cardiac injury (*P* < .001).^62^

## 5 CONCLUSION

Literature to date shows a clear correlation between cardiovascular disease and COVID‐19 severity with hypertension and diabetes the most prevalent comorbidities associated with adverse cardiovascular outcomes. Cardiac manifestations are an important aspect of disease manifestation in COVID‐19 with atrial fibrillation, myocarditis, heart failure, and cardiogenic shock the most common reported manifestations. The use of cardiac‐specific biomarkers (Troponin and NT‐proBNP) have shown a prognostic value with elevated levels linked to increased incidence of mortality, which can ultimately result in more rapid escalation of treatment. With a second wave expected by many experts to be worse than the initial wave, clinicians must be aware of the risk factors, the manifestations, and the investigations that could help in preventing such predictions becoming reality.

## CONFLICT OF INTERESTS

The authors declare that there are no conflict of interests.
